# Loop-Mediated Isothermal Amplification (LAMP): The Better Sibling of PCR?

**DOI:** 10.3390/cells10081931

**Published:** 2021-07-29

**Authors:** Marianna Soroka, Barbara Wasowicz, Anna Rymaszewska

**Affiliations:** Department of Genetics and Genomics, Institute of Biology, University of Szczecin, 3c Felczaka St., 71-412 Szczecin, Poland; marianna.soroka@usz.edu.pl (M.S.); anna.rymaszewska@usz.edu.pl (A.R.)

**Keywords:** LAMP method, isothermal amplification, SARS-CoV-2 detection

## Abstract

In 1998, when the PCR technique was already popular, a Japanese company called Eiken Chemical Co., Ltd. designed a method known as the loop-mediated isothermal amplification of DNA (LAMP). The method can produce up to 10^9^ copies of the amplified DNA within less than an hour. It is also highly specific due to the use of two to three pairs of primers (internal, external, and loop), which recognise up to eight specific locations on the DNA or RNA targets. Furthermore, the Bst DNA polymerase most used in LAMP shows a high strand displacement activity, which eliminates the DNA denaturation stage. One of the most significant advantages of LAMP is that it can be conducted at a stable temperature, for instance, in a dry block heater or an incubator. The products of LAMP can be detected much faster than in standard techniques, sometimes only requiring analysis with the naked eye. The following overview highlights the usefulness of LAMP and its effectiveness in various fields; it also considers the superiority of LAMP over PCR and presents RT-LAMP as a rapid diagnostic tool for SARS-CoV-2.

## 1. Introduction

One of the greatest achievements of molecular biology is the invention of the polymerase chain reaction (PCR) technique by Kary B. Mullis in 1983, for which he was awarded the Nobel Prize in 1993. An advantage of PCR is that it makes it possible to conduct genetic research even with small amounts of the targeted biological material. The technique can multiply any DNA fragment within two or even three hours. As a consequence of its many advantages and further modifications, PCR has become the fundamental tool in scientific, diagnostic, and forensic laboratory research [1,2,3,4,5]. Some of the PCR modifications improve its sensitivity (nano-PCR) or specificity (nested PCR); others allow for the real-time monitoring of product amplification (real-time PCR), can amplify very long DNA fragments (long PCR), or multiple fragments at the same time (multiplex PCR). All variants of PCR progress cyclically along stages with strictly determined durations and temperature conditions, comprising the denaturation of DNA, hybridization of primers into complementary DNA bases, and specific elongation of the DNA chain. This process requires special equipment (a thermocycler) and a considerable amount of time, which is needed not only for PCR itself but also for DNA/RNA extraction and the results visualization.

It should be noted here that polymerases have already been developed that are capable of synthesis even in the presence of inhibitors, which eliminates the need for the extraction of genetic material. For example, rTth polymerase has shown the ability to identify genetic material in the presence of inhibitors, depending on their concentration. This ability is higher for RNA than for DNA when both are used as biological material [6,7].

## 2. LAMP Method

In 1998, a Japanese company called Eiken Chemical Co., Ltd. designed a method known as the loop-mediated isothermal amplification of DNA (LAMP), eliminating certain difficulties native to PCR [8]. The technique is highly specific and increases the amount of amplified DNA even up to a billion copies over less than an hour, compared to a million copies yielded by the PCR. Isothermal amplification can be performed without advanced laboratory equipment, such as in a dry block heater or a water bath. Another innovative aspect of LAMP is its high specificity due to the use of several primers (from four to six), which can distinguish up to eight specific locations on the DNA template, compared to only two in typical PCR [9].

A deciding element responsible for the correct progression of the LAMP reaction is the primer design stage. Several pairs of primers must be optimized in terms of a range of factors, including concentration, location of nucleotide pairs, and distance between DNA regions. The primers must have a single-strand structure at 60–65 °C and must not create a stable double-strand structure. Using a bigger number of primers to amplify, the same sequence can increase the interactions between them. The design of LAMP primers can be carried out using online software such as PrimerExplorer (https://primerexplorer.jp/e/ (accessed on 24 July 2021)), LAMP Designer Optigene (www.optigene.co.uk/lamp-designer/ (accessed on 24 July 2021)), or Premier Biosoft (http://www.premierbiosoft.com/isothermal/lamp.html (accessed on 24 July 2021)). Furthermore, choosing the primers requires a prior analysis of the variation of many genomic sequences in the targeted species, depending on the LAMP goal. If such information is unavailable, sequencing must be applied to determine the variation of a given gene in the species, which considerably extends the time and effort needed for a routine LAMP application.

**Figure 1:**
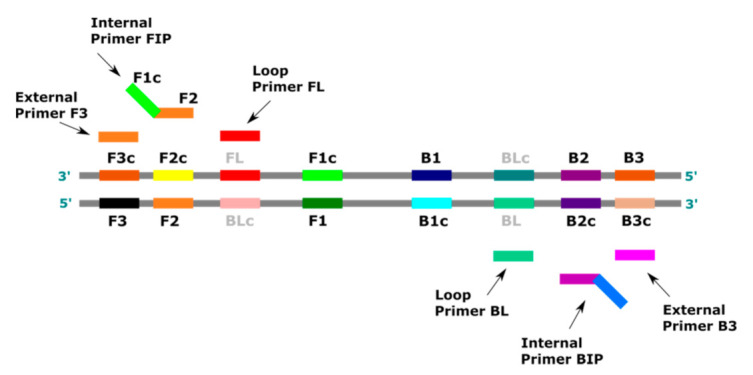
Primers used in the LAMP reaction. FIP (forward internal primer) contains a region F2 complementary to F2c of the matrix, and a free region F1c complementary to F1 on the newly-formed strand; BIP (backward internal primer) contains a region B2 complementary to B2c of the template, and a free region B1c complementary to B1 on the newly-formed strand; F3 (forward external primer) contains a region F3 complementary to F3c of the template; B3 (backward external primer) contains the region B3 complementary to B3c of the template; FL (forward loop primer) is complementary to the single-stranded loop between regions F2 and F1; BL (backward loop primer) is complementary to the single-stranded loop between regions B2 and B1. (Based on http://loopamp.eiken.co.jp/e/lamp/primer.html (accessed on 1 June 2021)).

The pairs of primers used in LAMP are as follows: the internal primers, forward internal primer (FIP) and backward internal primer (BIP); the external primers, forward primer (F3) and backward primer (B3); and the optional loop primers, loop primer forward (FL) and loop primer backward (BL) (Figure 1). The internal primers are long (45–49 bp) and complementary to two distant locations on the template (on the sense strand and the antisense strand). The external primers are shorter (21–24 bp) and are applied in lower concentrations in the reaction mixture to bind with the template more slowly than the internal primers. The internal and external primers, both forward and backward, combined with the Bst DNA polymerase, which shows a high strand displacement activity at 60–65 °C, create a dumbbell-like DNA structure [9].

This structure serves as a template for further amplification. Adding loop primers, which are complementary to the dumbbell-like DNA, increases the number of “starting points” during the LAMP reaction up to a total of eight amplified DNA sequences [9,10]. Thus, the loop primers significantly improve the efficiency and sensitivity of the reaction and reduce the time it takes by 50%. Furthermore, the loop primers activate only once when the artificial template has been created, which considerably increases the selectivity of the reaction [10].

**Figure 2:**
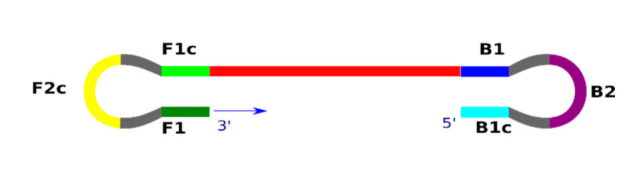
Schematic of a single-stranded DNA dumbbell-like structure, an artificial template for further amplification of the reaction using the LAMP technique. (Based on http://loopamp.eiken.co.jp/e/lamp/principle.html (accessed on 1 June 2021)).

The LAMP technique does not involve the DNA denaturation stage because, due to the aforementioned strand displacement activity of the Bst DNA polymerase, the reaction can be conducted in isothermal conditions [8,11]. All stages of LAMP are performed at a stable temperature of 60–65 °C, eliminating the need to use a thermocycler to precisely adjust the thermal and time profiles, which is necessary for the commonly-known PCR technique. The LAMP technique can be divided into three stages: the production of the starting material for the reaction, cyclic amplification, and elongation combined with recyclization (detailed figures and animation of principles can be found http://loopamp.eiken.co.jp/e/lamp/ (accessed on 26 July 2021)). The most important step in the first stage is to produce an artificial template in the form of single-strand DNA with a dumbbell-like structure (Figure 2).

The subsequent stages involve an exponential amplification of the self-priming template and the replacement of the strands, which yields a mixture of double-strand DNA. The final output of LAMP is cauliflower-like structures [8].

Over time, several variants of LAMP were introduced, including reverse transcription loop-mediated isothermal amplification (RT-LAMP), multiplex loop-mediated isothermal amplification (M-LAMP), and real-time observation of the product by modifying the original protocol, which allowed for RNA analysis and multiplex detection [12]. As with the RT-PCR variant, RT-LAMP uses reverse transcriptase combined with DNA polymerase to detect RNA sequences or polymerase with reverse transcriptase activity, e.g., Bst 3.0 or OmniAmp [13,14]. The procedure proved to be extremely helpful in diagnosing many RNA viruses [15,16,17]. In turn, M-LAMP can detect many pathogens in a single reaction or test tube if more starters or starters with unique fluorescence signals are used [18].

## 3. Detection of LAMP Products

A significant advantage of LAMP is the ability to detect products quickly using several methods. Every DNA amplification performed using PCR (except the real-time PCR) ends with electrophoretic separation of the products. However, preparing the gel (agarose or polyacrylamide) and the electrophoresis itself is both time-consuming, and visualizing the products requires dyes, many of which are mutagenic or carcinogenic. In contrast, the products of LAMP can be observed even with the naked eye immediately after the reaction ends or even while the reaction is running, without any additional procedures.

Many possibilities have been developed to detect positive LAMP reactions, such as colourimetric detection using fluorescent dyes, UV light irradiation, agarose gel electrophoresis, turbidity, real-time fluorescence, smartphone, lateral flow assay (LFA), and AC susceptometry. The fluorescent dyes calcein, the SYBR Green dyes, and the EvaGreen are the most widely used for detecting LAMP amplicons [12,19,20]. The other dyes used for this purpose are malachite green dye, hydroxynaphthol blue dye, Goldview dye, GelRed dye, SYTO fluorescent dye, leuco crystal violet (LCV) dye, and berberine dye [19]. All fluorescent dyes emit light of a specific length after binding with double-strand DNA except calcein, which forms a complex with magnesium obtained from LAMP reaction (see below). The results are observed under UV light irradiation and can be seen with the naked eye by changing colour, for instance, from orange to green in the calcein case. These methods can be combined with real-time analysis to allow for a concurrent quantitative assessment of the product [12,20]. However, real-time fluorescence monitoring requires costly equipment and proper supervision that is incompatible with fast, rapid, and cheap tests [19].

An extremely fast method that does not require advanced equipment is turbidimetric analysis [21]. The side product of LAMP, magnesium pyrophosphate (Mg_2_P_2_O_7_), allows the results to be assessed based on the opacity of the reaction solution or the presence of sediment. A discernible opacity indicates the presence of the product, whereas if the solution is clear, the product is absent. The sediment forms while the subsequent nucleotides are being added to the elongated DNA chain. During this synthesis, magnesium pyrophosphate reacts with the magnesium ions present in the reaction buffer, creating the sediment. The test tube becomes discernibly opaque once the concentration of magnesium pyrophosphate exceeds 0.5 mM. Such an analysis is impossible with PCR because the concentration of magnesium pyrophosphate is only about 0.02 mM. Furthermore, the temperature of PCR, or specifically, the denaturation taking place at about 95 °C, causes the pyrophosphate ions to hydrolyze into phosphate ions. The opacity of the solution allows for a qualitative assessment of the product. For a quantitative assessment, LAMP can be combined with real-time analysis. The yield of magnesium pyrophosphate is then compared to the yield of DNA [12].

Another technique of detecting a positive LAMP reaction is AC susceptometry, a very analytical sensitivity system able to recognize 1 attomolar (aM: 10^−18^ moles per litre) synthetic oligonucleotides of studied pathogens within 27 min. LAMP results are measured by a portable AC susceptometer, where the changes of hydrodynamic volume are estimated in the presence of streptavidin-magnetic nanoparticles (streptavidin-MNPs) mixed with LAMP reagents [22]. In addition, LFA is a favoured method because it is user-friendly, practical for in-field applications, lightweight, portable, and low-cost. Lateral flow assays coupled with the LAMP/RT-LAMP reaction take a total of 35–40 min for the molecular detection of pathogens [19].

A positive result of the LAMP reaction can also be checked using traditional electrophoresis in agarose gel. In contrast to the image obtained with PCR, the result of the LAMP reaction is visible in the form of bands of various lengths. The reason for such an image is the different lengths and structures of the obtained DNA products. However, visualization of LAMP amplicons by gel electrophoresis is time-consuming and may increase the risk of cross-contamination between samples due to the large amounts of DNA amplicons generated during the LAMP assay [19].

## 4. LAMP Application

**Figure 3:**
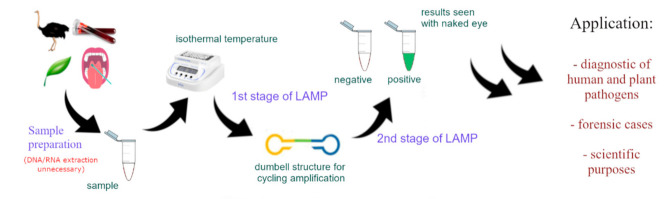
LAMP method process diagram from the sampling to the stage of visualisation of the results.

The LAMP technique greatly reduces analysis time, which is why it has come to the attention of diagnostic laboratories. The applicability of LAMP in the diagnostics of contagious diseases in both humans and domesticated animals continues to expand [16,21]. As a result of its high sensitivity, the technique can even identify microorganisms based on minuscule samples (10^−15^ g) [23]. The widespread application of LAMP in laboratories is possible due to the availability of primers on the market tested for the detection of viruses, bacteria, fungi, and parasites causing diseases in humans (e.g., COVID-19, SARS, hepatitis B, tuberculosis, and amoebiasis), animals (e.g., H5N1 influenza and aphthous fever), and useful and decorative plants [15,22,24,25,26,27] (Figure 3).

### 4.1. Detection of Plant Pathogens

An important application of LAMP, due to financial considerations, is the diagnosis of plant pathogens. The first study on the detection of plant diseases was published by Fukuta et al. nearly two decades ago, in 2003 [28]. The study concerned the Japanese yam mosaic virus (JYMV) and was conducted using RT-LAMP, which allows for quick, simple, and very sensitive identification of the RNA of plant viruses, as well as viroids, fungi, bacteria, and oomycetes [15,26,29,30,31,32,33,34,35]. The speed and specificity of LAMP-based tests mean that they can be performed even on-site to roll back a contaminated product immediately and prevent the pathogen from spreading. This can minimise disruption in trade caused by quarantine measures and allow the simultaneous application of non-quarantine disease control measures [36].

Molecular assays developed to date, based on the LAMP technique for detecting plant viruses, are 100 to 1000 times more sensitive than the conventional PCR assays of equivalent specificity [37,38,39]. The LAMP method also has the advantage of tolerating compounds that inhibit the standard PCR method [40]. LAMP assays described in subject literature can detect less than ten copies of the target sequence, which is close to the sensitivity of an optimized real-time PCR assay [8,41].

Another significant target are GMOs, for which the LAMP technique has proven highly effective [42]. A procedure was designed to quickly extract DNA from genetically modified plants employing mechanical maceration of the tissue in water. This means that the time-consuming stage of purification, necessary in PCR, was omitted; and despite the omission, the LAMP genotyping yielded positive results, allowing the procedure to be performed even in the field [43].

The identification of plant species using LAMP has also found application in herbalism. Identifying herbs based on their morphology or observation with a microscope are fairly often insufficient. There are many publications on the use of LAMP in the analysis of medicinal plants, including a study on distinguishing between ginseng and similar plants with no medicinal properties [44,45].

### 4.2. Detection of Animal Pathogens

Many papers demonstrate the effectiveness of the LAMP method in the detection of animal pathogens. However, most of these papers concern economically and epidemically significant diseases [24,46,47,48,49,50].

Due to the severe overfishing of the seas and oceans, fish abundance is falling dramatically. According to the Food and Agriculture Organization of the United Nations, the number of overfished stocks worldwide has tripled over half a century, and now a third of the world’s assessed fisheries are exceeding their biological limits [51]. This state of affairs implies the need for impactful prevention and control measures to reduce fish losses and, consequently, to minimise the economic impact of diseases, especially on fish farm owners. The development of molecular methods has made accurate detection of various pathogens possible. Standard molecular methods such as PCR and RT-PCR are time-consuming and require specialised equipment and personnel. The LAMP and RT-LAMP technique is an excellent solution; it can be used in the field, its specificity is sometimes higher than that of PCR, and analysis can be twice as fast [52,53].

Another economically important case is the detection of the FMD (foot-and-mouth disease), which may be fatal to cloven-hoofed animals [54]. For the purposes of a rapid detection of FMD virus (FMDV), the RT-LAMP protocol was developed to detect its RNA in less than an hour. The protocol duplicates a fragment of the virus’s 3D RNA polymerase gene, and the result can be observed with the naked eye [55]. The short analysis time allows rapid detection of diseased individuals that could infect an entire farm, leading to severe costs. In addition to economic considerations, the LAMP method can also be used to understand epidemiological issues, which are essential not only for animals but also for humans. One example is echinococcosis, a disease caused by *Echinococcus multilocularis*, found in foxes, raccoons, and rarely dogs and canids. Humans are the intermediate hosts and are mainly infected by eating berries with parasite eggs attached, transmitted directly by the infected animals or their faeces. The PCR technique demonstrates high specificity in detecting the parasite’s genetic material, but this is rarely used due to its complexity and cost. Consequently, researchers described an assay based on the LAMP method that detects the mitochondrial gene *nad1* [56]. The sensitivity of this assay was very similar to PCR, but LAMP omitted the step of extracting the genetic material. Furthermore, LAMP had other advantages over PCR, especially in terms of analysis time, complexity, and field applicability [56].

### 4.3. Application of LAMP in Forensics

A frequent issue in forensic investigations is determining whether the material collected at the crime scene is human or animal in origin. Forensic genetics is even divided into human forensic genetics (HFG) and non-human forensic genetics (NHFG). Non-human genetic material constitutes an auxiliary source in forensic investigations and is used fairly commonly in court cases. The analyses are performed to differentiate between plant and animal species and identify microbiomes, as well as in cases involving wildlife crimes, bioterrorism, and food [57]. To help determine the origin, a test was designed combining LAMP and a colourimetric reaction with nanoparticles of gold [58]. The primers designed for the test recognized eight regions of the human cytochrome b, and the specificity of the reaction was tested based on the results obtained for 11 animal species, including species closely related to humans, i.e., the chimpanzee and the orangutan. The results of LAMP were determined by observing changes in the colour of the solution. The method proved to be a reliable, specific, and inexpensive forensic tool due to its ability to identify human traces. The standard immunological tests for detecting human blood can yield a positive result for primate and mustelid (weasel and badger) blood [59,60].

The LAMP technique has also found application in commercial offences against consumers. The food industry suffers from frauds, whereby manufacturers declare false compositions of their meats and meat products. Food frauds impact consumers for many different reasons; for example, there are religious tenets that forbid the consumption of pork and pork products, for instance, in Judaism or Islam. Some products of animal origin, such as albumin from porcine plasma, are potent allergens. Meat prices vary depending on the country of origin and the age and sex of the animal. An assessment of the composition of meat products sold in stores and at marketplaces in Istanbul (Turkey) found that 53.4% of the cases were fraudulent [61]. The information provided on the labels did not match the real composition of the products. Such practices have led to an interest in simple, specific techniques to identify food in the field, with no special equipment or training. Such assessments usually involve the analysis of mitochondrial genes, i.e., cytochrome b (*cytb*) and cytochrome c oxidase subunits I and II (*cox1* and *cox2*), and the control region (CR) [62]. Food frauds are especially prevalent in poor and developing countries with high populations, where the demand for meat and meat products is rising, which inflates their prices [63]. Subject literature contains an increasing number of reports on pork frauds and their detectability with LAMP [64,65,66,67,68].

The most common targets of fraud are pork and beef products [63]; however, not enough tests have been designed to date. Some of the available tests can detect pork at a concentration of 0.01% in the mixture, which corresponds to 0.1 g of pork in 1 kg of meat [64,69,70]. Another test can determine the composition of bovine tissues in mixtures with closely related species, i.e., buffaloes, goats, and sheep. The results of the test have demonstrated that LAMP is also highly specific and effective at identifying bovine tissue, being able to detect it in mixtures of meat from two species at a minimum concentration of 0.01 ng [63]. Other popular targets of fraud include ostrich meat. It has good nutritional value and resembles chicken in terms of composition and beef in terms of taste. A majority of ostrich meat on the European market is imported from Africa. There are two ways in which such a meat fraud can proceed. Firstly, the meat may come from the protected species *Struthio camelus* rather than the domesticated *S.c. domesticus* (the African Black ostrich). Secondly, ostrich meat can be substituted with the cheaper meat of domestic animals. Abdulmawjood et al. [71] designed a method for detecting the authenticity of ostrich meat with the LAMP technique. The test is based on the assessment of cytochrome b and has proven sensitive and accurate enough to identify meat on-site, for instance, in a restaurant or a store. It is worth underlining that the test works regardless of whether the meat was thermally processed, flavoured (with salt, spices, or oils) or canned. Furthermore, research indicates that the test can detect a 0.01% admixture of ostrich meat, which corresponds to 0.1 g of ostrich meat in 1 kg of a meat product. The test takes 15–20 min, from sampling to the results [71].

Wang et al. [72] obtained interesting results in their analysis of the origin of starch in traditional Chinese noodles made from sweet potatoes (*Ipomoea batatas*). The noodles are very popular among consumers, which encourages fraud. Based on ITS sequences, the LAMP test showed that as many as 57.7% of the products containing sweet potato noodles sold in retail in China had starch from the much cheaper cassava (*Manihot esculenta*) added to them. Some of the less commonly used substitutes are corn starch (*Zea mays*) and potato starch (*Solanum tuberosum*). The method designed by Wang et al. [72] proved to be precise and specific to all natural sweet potato starch substitutes. Furthermore, the application of the ITS sequence, which has multiple copies in the genome, increases the sensitivity of real-time LAMP. The simplicity, speed, and high specificity of the tests allow them to be used by food quality control offices to protect the consumers’ rights and preferences from fraud [72].

For human samples, LAMP can be used to identify body fluids. In 2019, Jackson et al. [73] presented the results of an application of RT-LAMP in combination with a simple optical method of detection and dedicated software running on a smartphone. The assessment concerned all body fluids, i.e., blood, saliva, vaginal discharge, semen, and azoospermic semen. A unique mRNA marker was selected for each fluid based on tissue specificity. The markers comprised human β-globin (HBB) for the blood, human beta-defensin (HBD-1) for the vaginal discharge, human semenogelin 1 precursor (SEMG1) for the semen, and histatin 3 precursor (HTN3) for the saliva. The amplification took 15 min for the blood and 30 min for the semen, saliva, and vaginal discharge. Furthermore, the study confirmed once again that the presence of PCR inhibitors (e.g., heme or indigo dye) did not affect the course of RT-LAMP. Jackson et al. [73] used LAMP to create a body fluid panel, which is a simple and effective test that can be performed without additional equipment or training. A further innovation is the use of menstrual blood in addition to the aforementioned fluids. In forensic investigations, the ability to determine whether the blood sample found at the crime scene comes from venous blood or menstrual blood is very important, as in many cases it can aid arrival at the correct conclusion. Notably, the method does not destroy the DNA evidence, which can also be used for profiling. The study constitutes the first step towards an innovative approach to identifying body fluids for forensic purposes [74].

In 2008, LAMP tests were performed to identify human sex based on the amelogenin gene located on the X and Y chromosomes [75]. The genetic material was obtained from teeth stored at room temperature for between 1 and 25 years. Furthermore, Kanchanaphum [76] proposed LAMP and LAMP-lateral flow dipstick (LAMP-LFD) tests, in which the *SRY* gene was the desired sequence, and the results were compared to those obtained using traditional PCR. The DNA was extracted from bloodstains collected from various surfaces (fabric, wood, clay, and tiles) and stored at room temperature for 1, 7, 30, and 60 days. The PCR technique did not provide satisfactory results for the 30-day samples. Conversely, LAMP yielded a positive result for all male samples. In addition, in combination with the lateral flow device (LFD) technology, LAMP is more sensitive than traditional PCR and does not require complicated equipment. The procedure involves simply dipping the LFD band in an LFD buffer, waiting 5–10 min, and determining the result with the naked eye [76]. The undeniable advantage of the LAMP-LFD combination is the equipment-free procedure and the short testing time and applicability in field research.

### 4.4. Detection of Human Pathogens

Providing quality healthcare for infectious diseases depends on how effectively and how quickly the responsible pathogens are detected in samples. Over the years, many molecular tests have been developed to enable their rapid and sensitive detection and identification. However, these methods, although reliable and efficient, require expensive equipment, reagents, and trained personnel. The LAMP method has also been the focus of interest for scientists working on pathogens that threaten human life and health. One such example is the infectious periodontal disease caused most commonly by the three bacteria *Porphyromonas gingivalis*, *Bacteroides forsythus*, and *Treponema denticola* [77,78,79]. It showed a strong correlation between mixed infections caused by *P. gingivalis*, *B. forsythus*, and *T. denticola*, and periodontitis in adults [79]. In addition, the bacteria are responsible for the development of halitosis [80]. A system for accurate and rapid diagnosis of periodontal disease is essential in periodontal treatment, especially due to its proven association with cardiovascular disease and atherosclerosis [81,82,83]. One of the most commonly used techniques to diagnose such infectious diseases is PCR [84]. However, as has already been mentioned, it is a method requiring advanced equipment. Consequently, the first test based on the LAMP method was developed as early as 2005. It enables faster and simpler detection of this type of pathogens [85]. Due to the modified protocol, the LAMP method allows for a qualitative and quantitative analysis of infectious disease pathogens, which is necessary for an accurate, detailed diagnosis [12].

RT-LAMP has been developed for influenza viruses [86,87,88], the dengue virus [89,90], respiratory syncytial virus [89,91], hepatitis C virus [92,93], Ebola virus [94], Zika virus [19,95,96], and Middle East respiratory syndrome coronavirus (MERS-CoV) [97,98]. One of the most critical applications of this method is detecting HIV, which can be identified even in its dormant form thanks to RT-LAMP [99,100,101].

## 5. LAMP for SARS-CoV-2 Detection

The outbreak of the SARS-CoV-2 pandemic in March 2020 forced scientists to redouble their efforts to design optimal screening tests. The most important considerations in detecting dangerous pathogens are testing speed and simplicity. Tests based on PCR are expensive, require specialized laboratories, take a few days, and are vulnerable to delays and shortages in reagent deliveries. Antigen tests, which involve the binding and detection of the surface proteins of a virus, are quick and inexpensive but yield a high percentage of false negatives. Many immunoenzymatic methods are insensitive and non-specific. Moreover, they take 7–14 days to detect antibodies and do not distinguish between acute and past infections. Consequently, tests based on RT-LAMP seem to be an effective method for detecting an active COVID-19 infection. Because RT-LAMP tests require little equipment, they can be performed in non-laboratory conditions, e.g., at the airport or in small hospitals and other medical facilities with no access to diagnostic laboratories.

Scientists have collaborated with biotechnological companies to create several assays for quick detection of SARS-CoV-2 (an overview in Table 1) [26,102,103,104]. The assays use reverse transcriptase to amplify cDNA based on the RNA of the virus, as with RT-PCR. They can detect even low concentrations of the virus in the sample, and preparing the samples for testing takes only a few minutes.

The first assay for the detection of SARS-CoV-2 was designed shortly after the outbreak, which indicates that the technique has good potential [103]. The subsequent RT-LAMP assays were designed to detect several key areas in the virus’s genome, including the ORF1ab, and S and N genes. The ORF1ab gene is responsible for replicating the viral genome [105]. The S gene takes part in binding SARS-CoV-2 with the human ACE2 protein [106]. Lastly, the N gene encodes the nucleocapsid protein, which is conservative in most coronaviruses [107,108,109].

Yu et al. designed the iLACO assay, which identifies amplicons based on the observation of fluorescence [17]. The assay involves detecting the ORF1ab gene. Despite its slight complication, the assay is much faster than conventional RT-PCR, taking 15–40 min to complete, depending on the number of RNA copies in the sample. The green SYBR dye was used to strengthen the fluorescence resulting from the intercalation of the dye within dsDNA. In order to improve sensitivity for samples with a small number of viral RNA copies, the assay also used the blue GeneFinder dye.

Another assay is the barcoded RT-LAMP protocol, named Lamp-Seq [110]. In this assay, the FIP primer is labelled with a compressed barcode sequence of 10 nucleotides, which does not affect the efficiency of the reaction in any way. After 30 min of standard RT-LAMP analysis, each sample is heated for 10 min at 95 °C to terminate the reaction. Next, the samples are combined into pools of 1000–10,000 samples each. A twelve-stage PCR is performed for each pool using the barcode FIPs to label those containing the inserted sequence. The obtained products are sequenced, and the barcodes bound to the viral sequence are detected computationally to identify an individual with an active infection. The protocol authors provided precise optimal parameters for their method and estimated that if 1.3% of a population is infected with SARS-CoV-2, then total false positives and false negatives will amount to less than 0.2%. The authors also stated that the test would cost 7 USD per sample. The protocol would allow for the screening of a million individuals per day using modern tools, such as next-generation sequencing [110].

Another modification of RT-LAMP for SARS-CoV-2 detection is the two-stage Penn-RAMP method, which involves recombinase polymerase amplification (RPA) followed by the LAMP reaction [111]. The first stage takes 20 min at a temperature of 38 °C in the presence of recombinase, which makes it easier for the F3 and B3 primers to locate the targeted sequence. Next, the mixture is added to LAMP reagents at a ratio of 1:9. The reaction takes 40 min at 65 °C. According to some reports, Penn-RAMP has the highest sensitivity among all the RT-LAMP and RT-PCR methods that have been described to date [48]. The limit of detectability is seven copies of viral RNA per reaction. Furthermore, RPA is resilient to inhibitors and limits the yield of false amplicons, as confirmed in clinical trials for HIV [111].

Scientists were also able to combine LAMP with CRISPR-Cas12 [112]. This method, called the SARS-CoV-2 DNA endonuclease-targeted CRISPR trans reporter (DETECTR), uses the Cas12 enzyme following the RT-LAMP procedure to detect specific sequences of the E and N genes and separate the previously created structure. The N-oriented (nucleoprotein-oriented) DETECTR assay exclusively detects the SARS-CoV-2 virus, and the E-oriented assay (envelope-oriented) detects the following viruses: SARS-CoV-2, SARS-CoV, and bat-SL-CoVZC45. The detection takes places through the FAM-biotin reporter and lateral flow bands designed to identify labelled nucleic acids. The authors of the assay believe that it can also be useful for multiplexing.

There are few RT-LAMP tests that amplify several SARS-CoV-2 genes at the same time. One such test uses primers to detect the genes encoding the RNA-dependent RNA polymerase (RdRP) and envelope (E) and nucleocapsid proteins (N) [113]. The efficiency of the tests was compared in a clinical setting with direct RT-qPCR methods using the Seegene Allplex^TM^ 2019-nCoV test and the Kogenebiotech PowerChek™ 2019-nCoV Real-time PCR assay. The analysis provided comparable values for both methods in the former, whereas multiplex RT-LAMP showed higher sensitivity and accuracy in the latter case.

A very interesting and simple test is the single-stage RT-LAMP for use at home [114]. It was performed using ovens with a “keep warm” function to maintain a stable temperature. The test only required opening the tube one time to place the sample inside. Considering the growing need for extended screening tests, the at-home RT-LAMP method opens up a new path in molecular diagnostics. Because all stages are simple enough to be performed without training and user engagement is minimal, the testing possibilities seem limitless. The concept of at-home molecular diagnostics also lends itself well to detecting other contagious diseases. However, no data are available about the real performance of such DIY (do it yourself) assays.

Another example of a DIY assay is the LAMP-enabled rapid test (ALERT) [115]. According to its designers, the test can easily be performed at home and care centers, as well as in laboratories conducting large-scale analyses. The assay is inexpensive enough (>5 USD) to be affordable for everyone. Users can collect the sample, isolate the viral RNA, conduct the RT-LAMP reaction, and visualize the results all by themselves over 60 min of a simple, five-stage protocol with high sensitivity (0.1 to 2.0 viral molecules/µL) and specificity (>97%). The ALERT is exceptional in that it implements QUASR reporting to confirm the presence of viral RNA, which reduces the rate of false positives and enables multiplex analyses. The designers underline that the assay does not have to be stored at a low temperature, which is important for at-home tests [115].

The RT-LAMP tests for detecting SARS-CoV-2 display a 100% or near-100% specificity, yielding negatives for other viruses of the respiratory systems, including MERS, MHV, and BtCoV, as confirmed by comparative analyses between the sequences of SARS and other coronaviruses [103,116]. No mismatch was observed for SARS-CoV-2. For common coronaviruses, the mismatch amounted to 27–54% [103]. In patients with increased viremia, detection is faster and sensitivity increases, reaching the maximal value for samples obtained from the nasopharynx. The test sometimes takes longer than 30 min; nonetheless, shortening the testing time to 60 or even 90 min still constitutes fast detection [117]. It should be noted that while the specificity of the reaction performed by various research centers with the inclusion of the isolation stage reached 100%, sensitivity was lower than with RT-qPCR when the extraction of genetic material was omitted [102].

In order to eliminate false negatives results obtained with RT-LAMP, the protocol can be preceded by a sample preparation stage. In addition to improving sensitivity, preparing the samples decreases the risk of infection compared to the standard extraction method. Preparation involves disintegrating the virions, releasing the viral RNA, deactivating RNases using the appropriate detergents, a temperature of 95 °C, and purification in a silica suspension [118]. The study was performed with samples collected from the nasopharynx and saliva, and the RT-LAMP products were observed based on changes in colour and the detection of fluorescence. The obtained results showed that RT-LAMP is insensitive to the applied detergents (up to a concentration of 3%); furthermore, the RNases inactivation step improved sensitivity from 100 to 50 copies of viral RNA/µL, and the purification stage (in a silica suspension) improved it even down to 1 copy/µL [118].

Saliva, sputum, nasopharynx swabs, and blood are all viable sources of material for CARS-CoV-2 detection. However, the best RT-LAMP results were observed for the nasopharynx swabs, which have the highest viral RNA content. Research indicates that for blood, the maximum volume that can be added to 50 µL of the reaction mixture to obtain a satisfactory level of amplification is 5 µL [119].

Currently, the gold standard for the molecular diagnosis of COVID-19 is RT-qPCR. However, many researchers are investigating RT-LAMP as a potential substitute. While RT-qPCR is very sensitive and specific, it requires expensive equipment and experienced laboratory staff to perform the test and interpret its results. Even with portable equipment, the cost and time of the analysis make RT-qPCR impossible for rapid and massive detection of SARS-CoV-2, especially in countries that cannot afford to pay for the tests and in locations too distant from laboratories. In turn, despite its numerous advantages, the precise sensitivity of the different RT-LAMP tests is still undetermined due to an insufficient number of studies conducted with large clinical sample sizes (main features compared in Table 2).

Considering that many promising genetic tests for the detection of SARS-CoV-2 based on RT-LAMP are currently available, it is time to use them in commercial diagnostics, a service that is offered by some countries; even though many publications indicate the high sensitivity and specificity of RT-LAMP methods, these methods are still applied primarily as screening tests and are considered incomplete. The UK has set a good example of the use of RT-LAMP tests in diagnostics. According to the British Medical Journal, as many as 50% of the tests performed using the Rapid Test RT-LAMP method (OptiGene, Horsham, UK) yielded a false negative in a pilot study conducted in Manchester prior to their introduction in the Liverpool health care system [120]. Studies were also conducted in Southampton, Basingstoke, and Salford. To date, the UK government has spent as much as 358 million euros on purchasing 90 million twenty-minute tests and 600 Genie HT machines (OptiGene) [121]. All purchases were made as part of Operation Moonshot, the primary goal of which was to conduct mass testing among the population. The initial plans involved testing 10% of the population of England per week. A month after the pilot study has begun, the Guardian reported that the tests were able to detect only 46.7% of total infections in Salford and Manchester [122]. In turn, according to the scientists who took part in Operation Moonshot, the sensitivity of the tests amounted to 96% for individuals in the infectious stage [120].

British scientists have pointed out that mass testing generates a high number of false positives [123]. Even if the tests were 99% specific, 1% of the population would still have received a false result. In the UK case, this would amount to 600,000 individuals, including individuals who had contact with them. Estimates showed that the number of false positives could exceed the number of actually infected individuals at a 1000-to-1 ratio [124], and as much as 41% of the UK population would have to undergo unnecessary self-isolation within six months, which would result in school closings and a loss of income for many [125,126,127]. According to the UK governmental website, the OptiGene RT-LAMP assay shows a sensitivity of 79% and a specificity of over 99% [128].

Currently, RT-LAMP tests are primarily used by tourism and sports companies and corporations. For instance, the FRANKD test manufactured by GeneMe (Poland) is used by the Virgin Atlantic Airlines and the Lewes football club from East Sussex. Conversely, the RT-LAMP Duo assay test (Genomtec, Poland) was purchased toward the end of March 2021 by a partnership of an independent group of Selectour (GIEASHA) travel agencies [129]. The collaboration was motivated by the fact that the RT-LAMP-based test perfectly identifies the Breton variant of SARS-CoV-2, in contrast to RT-PCR. The partnership also contacted a research hospital in Warsaw to conduct a comparative trial on saliva samples between direct-RT-LAMP and RT-PCR as a reference method. [129]

The trials for the other RT-LAMP tests are being conducted throughout the world in similar circumstances. Consequently, the results of comprehensive clinical trials involving RT-LAMP can be expected to arrive this year.

As a result of the epidemic, the LAMP method is under improvement, along with the commonly used RT-PCR. Although it is already treated as the gold standard, modifications are constantly in progress. Such modifications can significantly improve the diagnostic process by simplifying procedures, shortening the time before the results arrive, or mitigating the effects of reagent shortages. Consequently, ready-to-use kits for RT-qPCR in one hour have already been developed (MediPAN-COVID+Flu, Poland). A vital modification, which significantly shortens the analysis, involves the use of mastermixes insensitive to inhibitors. The best example is the extraction-free, multiplexed amplification of SARS-CoV-2 RNA described by Byrnes et al. [130]. The tests were performed on 246 clinical samples, resulting in 86% sensitivity and 100% specificity. The described protocol uses the CDC (Centers for Disease Control and Prevention, Atlanta, GA, USA) singleplex targets and has a limit of detection (LoD) of 2 copies/μL [130].

## 6. Limitations of the LAMP Method

A disadvantage of LAMP is its sensitivity to cross-contamination, i.e., material present in the aerosol. Consequently, it is recommended that rooms be ventilated, and different samples be analysed separately. For obvious reasons, this may not always be possible. Another disadvantage of LAMP is that it is difficult to check the samples for the presence of reaction inhibitors, as this requires two reactions, one to detect the inhibitors and the other to amplify the material. Moreover, while LAMP is a superb diagnostic tool, its products cannot always be used for further analyses, such as cloning or sequencing [16]. The target products of the LAMP reaction are short, and as such, any contamination of the sample with exogenous genetic material may impact the outcome. Persons performing the test should be aware of the risk of contaminating the samples and follow special sterility procedures.

The significant limitations of the RT-LAMP method and its use in the diagnosis of SARS-CoV-2 are not the lack of developed tests, but rather the bottlenecks that significantly limit its applicability. Of all the 32 LAMP tests described by researchers worldwide, only seven originate from Europe, and only two have been tested on biological material [131]. How many public scientific institutes have described a LAMP-based SARS-CoV-2 diagnostic kit that is already commonly used? The vast majority of RT-LAMP kits on the market are developed by private scientific institutions or biotech companies. In order to bypass the possible bottlenecks that paralyse LAMP’s diagnostic possibilities, countries’ governments should allocate funds for the creation of such national tests from the state funds, as was the case for the RT-qPCR kit developed by the Institute of Bioorganic Chemistry of the Polish Academy of Sciences (MediPAN-COVID+Flu), which was approved for commercial diagnostics, and financed by public institutions. As a result of the lack of interest in the method, scientists develop further kits, which do not meet the expectations of institutions fighting the SARS-CoV-2 epidemic. As mentioned earlier, RT-LAMP tests available on the market were developed by private entities and are used by the same. These tests deserve more extensive attention both in research (the improvement) and political consultation (the expressed expectations).

The case of the UK demonstrates that RT-LAMP, while extremely attractive, raises concerns about its practicality. Even though the entire world agrees that mass testing is the most important step in limiting the spread of SARC-CoV-2, no government is willing to accept the consequences of such an initiative. This is clearly visible in the number of tests that are granted certificates and enter the market. The methodology is rapidly developing; however, there are relatively few particular tests because nearly all commercial tests are single-gene, and in the case of RT-qPCR, double-gene. This means that a positive result needs to be confirmed with a complete test. In practice, the commercially available RT-LAMP tests are more expensive than antigen tests, and it usually takes more than 24 h for the results to arrive. This is not what scientists had in mind when they worked on designing a quick and effective diagnostic tool viable for mass testing.

It is important to emphasise the critical issue that the LAMP method is newer than RT-PCR and, as such, has not been researched nearly as extensively. Tests using RT-LAMP are still being assessed in clinical settings. Therefore, much practical information is lacking, i.e., realistic limits of sensitivity, reliability of the test, efficacy in untreated patient samples, and ensuring the validity of RT-LAMP in the field [131].

## 7. Summary

An undeniable benefit of the current interest in the LAMP technique is the optimization of primer design, an aspect that previously seemed difficult and discouraging, and the design of a fast and effective method for purifying the samples before analysis. The specificity of RT-LAMP has been complemented with tools such as CRISPR and high-throughput sequencing. As a result, the method can confirm the presence of viral sequences with optimum accuracy and can also be used for other types of analyses. Reports have also been published about specially manufactured cartridges and equipment connected to a smartphone to monitor the outcome of amplification. Even though researchers noticed the enormous potential of LAMP a relatively long time ago, the many new modifications and study results have now shown that LAMP, in addition to being an effective method for detecting pathogens, is also viable for forensic investigations and court cases, in which the time required to complete the analysis is often a key factor. The LAMP technique can be used to reveal contraband and prevent the illegal trade of animals and animal and plant products. It also seems to be the perfect answer to the rising demand for the molecular, diagnostic, and forensic laboratories. The usefulness and effectiveness of LAMP are demonstrated by a growing number of publications on the subject, and there are already so many possible applications and available tests that they vastly exceed the scope of this paper.

The wide availability of tested commercial assays for detecting human pathogens can provide a means of a quick diagnosis that can even be performed “by the patient’s bed”, especially in peripheral health care settings and private clinics. Likewise, LAMP can be used to identify plant pathogens and quickly quarantine the affected plant, thus avoiding significant financial losses. LAMP is also a beneficial and valuable tool for developing countries due to its ease of use, without the need for sophisticated equipment or experts. The case of the SARS-CoV-2 epidemic demonstrated that commercial innovations in PCR-based diagnostics aimed at increasing the efficiency of high-throughput screening were concentrated exclusively in centralised facilities. Such developments exacerbate the already restrictive costs for less affluent laboratories that aspire to adopt the molecular methods. As a result, PCR-based pathogen detection methods have been mainly limited to central laboratories in developed countries or private laboratories, which means that the potential benefits of these methods have not been fully realised. The growing popularity of LAMP in a wide range of fields suggests that it will soon become a gold standard, alongside PCR.

## Figures and Tables

**Table 1:** Overview of LAMP assays for diagnosis of COVID-19 reported in this review. Abbreviation: E—gene coding envelope protein, N—gene coding nucleocapsid protein, ORF—open reading frame, RdRP—gene coding RNA dependent RNA polymerase, S—gene coding spike protein. The symbol—means a lack of information.

Authors	Gene Target	Type of Samples	Number of Samples	Detection of Results	Limit of Detection (LoD)	Sample Preparation	Sensitivity/Specifity
Lamb et al. [103]	ORF1ab	Nasopharyngeal swab	60	Fluorescence detection, SYBR Green	3 copies/µL	RNA extraction	—
Dao Thi et al. [102]	ORF1a, N	Nasopharyngeal swab	95	WarmStart Colorimetric LAMP	—	5 min of hot swab–to–RT-LAMP assay	92–99.7%
Pang et al. [104]	N, E	Nasopharyngeal swab	100	Fluorescence detection, SYBR Green, GeneFinder	30 copies/µL	—	94%
Yu et al. [17]	ORF1ab	Nasopharyngeal swab	43	Fluorescence detection	10–100 copies/µL	RNA extraction	97.6–100%
Schmid-Burgk et al. [110]	ORF1a, N	Nasopharyngeal swab	28	Deep sequencing	100 copies/µL	Unpurified or lysed swab sample	—
El-Tholoth et al. [111]	ORF1ab	Synthetised	—	Fluorescence or colorimetric detection, LCV dye	7 copies/µL	Eluting swab into water	100%
Broughton et al. [112]	N, E	Nasopharyngeal swab	78	Lateral flow assay (LFA)	10 copies/µL	RNA extraction	95–100%
Jang et al. [113]	RdRP, N, E	Nasopharyngeal and oropharyngeal swabs, sputum, saliva and urine	292	Fluorescence detection	10 copies/µL for N and RdRP genes: 100 copies/µL for E gene	RNA extraction	RdRP: 93.9%, N: 94.6%, RdRP/N: 96.9%
Lei et al. [114]	—	Synthetised	—	Turbidity and fluorescence detection, SYBR Green	48 copies/µL	Eluting swab into water	—
Bektaş et al. [115]	N	Nasopharyngeal swabs, nasal mid-turbinate swabs, nasopharynx flush through	—	Fluorescence detection, SYBR Green	0.1–2 copies/µL	Quick RNA extraction	>97%
Huang et al. [116]	ORF1ab, N, S	Nasopharyngeal swab	16	WarmStart Colorimetric LAMP	0.8 copies/µL	Eluting swab into water	100%
Chow et al. [117]	ORF3a, E	Nasopharyngeal swab, sputum, throat swab	223	WarmStart Colorimetric LAMP	~2 copies/µL	RNA extraction	95.07–98.21%
Rabe et al. [118]	ORF1a, N	Nasopharyngealswab, saliva	—	WarmStart Colorimetric LAMP	1 copie/µL	Simple inactivation/ lyse step	85%
Wang et al. [119]	N	Synthetised	—	Fluorescence detection, EvaGreen	6 copies/µL	Extraction step omitted	—

**Table 2:** Comparison of the main features of LAMP and PCR techniques.

	LAMP	PCR
Temperature	Isothermal reaction (60 to 65 °C)	Thermal cycling (multiple heating from 45 °C to 98 °C)
Reaction time	<1 h	~2 h
DNA extrac-tion	Not required	Required
Primers	4–6 primers recognize to 6–8 targets, extra looping primers increases sensitivity and effectiveness	2 primers recognize 2 targets
Equipment	Dry block heater/water bath	Thermocycler
Modifica-tions	Real-time LAMP, MP-LAMP, RT-LAMP	Real-time PCR, MP-PCR, RT-PCR, nested PCR, nano-PCR, long PCR, RFLP-PCR
Sensitivity	100× higher than standard PCR 100× lower than nested PCR	Up to modification
Products de-tection	With naked eye: turbidimetric analysis, fluorescent detection, electrophoresis, real-time protocol	Electrophoresis, real-time protocol

## Data Availability

Not applicable.
